# Nogo receptor–vimentin interaction: a novel mechanism for the invasive activity of glioblastoma multiforme

**DOI:** 10.1038/s12276-019-0332-1

**Published:** 2019-10-24

**Authors:** Yun Hee Kang, Seung Ro Han, Hyungtaek Jeon, Suhyuk Lee, Jisu Lee, Seung-Min Yoo, Jong Bae Park, Myung-Jin Park, Jong-Tae Kim, Hee Gu Lee, Myung-Shin Lee, Seung-Hoon Lee

**Affiliations:** 1 Eulji Biomedical Science Research Institute, Eulji University School of Medicine, Daejeon, 34824 Korea; 2 Department of Microbiology and Immunology, Eulji University School of Medicine, Daejeon, 34824 Korea; 3 Specific Organs Cancer Branch, Research Institute and Hospital, National Cancer Center, Koyang, Korea; 4 Divisions of Radiation Cancer Research, Research Center for Radio-Senescence, Korea Institute of Radiological and Medical Sciences, Seoul, Korea; 5 Immunotherapy Convergence Research Group, Korea Research Institute of Bioscience and Biotechnology, Daejeon, 34141 Korea; 6 Department of Neurosurgery, Eulji University School of Medicine, Daejeon, 34824 Korea

**Keywords:** CNS cancer, Cell invasion

## Abstract

Nogo receptor (NgR) has been shown to inhibit the migration and invasion of human glioma cells. However, little is known regarding the regulatory mechanisms of NgR in glioblastoma multiforme (GBM). In this study, we propose a novel mechanism that regulates the maturation process of NgR through an interaction with vimentin. The inhibition of TGFβ1 activity by LY2109761 attenuated the migration/invasion of GBM cells by upregulating cell-surface NgR. Conversely, the treatment of GBM cells with TGFβ1 suppressed NgR maturation. We showed that NgR and vimentin interact, which could be a possible mechanism for the suppression of NgR maturation. The knockdown of vimentin suppressed the migration/invasion of GBM cells through the increased maturation of NgR. Finally, TCGA (The Cancer Genome Atlas) analysis also supported the association of NgR and vimentin. The maturation of NgR is regulated by the interaction of vimentin and NgR, which attenuates the invasive activity of GBM, and might be a potential therapeutic target for brain cancer.

## Brain cancer: A possible target to stop spread

A mechanism that prevents the maturation of a protective cell surface protein during the spread of brain cancer could be a therapeutic target. Aggressive glioblastoma multiforme tumors spread quickly, lowering survival chances. The transforming growth factor-beta 1 (TGFβ1) protein is implicated in the rapid spread of cancer cells through the brain’s white matter fibers. However, cancer spread can be limited by the mature form of a protein receptor called nogo receptor (NgR), which is expressed on white matter cell surfaces. Using human glioblastoma cell cultures, Seung-Hoon Lee and Myung-Shin Lee at Eulji University School of Medicine, Daejeon, South Korea, and co-workers demonstrated how the interaction between NgR and another protein enhances TGFβ1 pathway activity and prevents NgR maturing. When the team inhibited TGFβ1, the interaction was disrupted, allowing NgR maturation and preventing tumor spread.

## Introduction

Glioblastoma multiforme (GBM) is the most aggressive type of malignant primary brain tumor^1^. Despite multimodal treatment with surgery, radiotherapy, and chemotherapy, the prognosis of GBM is poor, with a median overall survival of 14 months and 2-year survival rates of <10%^2–4^. The failure of GBM treatment is attributed, in part, to the widespread infiltration of tumor cells into the normal brain parenchyma, leading to inevitable tumor recurrence, as well as the resistance of GBM to conventional therapeutics^5–7^.

Vimentin is a type III intermediate filament protein expressed in a wide range of cell types^8–11^ that has gained increasing attention as a characteristic biomarker for epithelial–mesenchymal transition (EMT)^12^. Vimentin can affect various biological behaviors of tumor cells^13–15^. Accumulating evidence indicates that vimentin is associated with the malignant progression of brain cancer^16–19^. However, the effect of vimentin on the Nogo-66 receptor, which contributes to the myelinated invasiveness of glioma, remains elusive.

Recently, a receptor for Nogo-66 (NgR) was identified^20^. NgR is a 473-amino acid protein (52 kDa) composed of a signal sequence, a leucine-rich repeat (LRR)-type N-terminal domain, eight LRR domains, a cysteine-rich LRR-type C-terminal-flanking domain, a unique C-terminal region, and a glycosylphosphatidylinositol (GPI) anchorage site^21^. The removal of 26 amino acids from the C-terminal region of NgR results in a 49-kDa mature form of NgR, as predicted by Swissprot. Three structurally unrelated myelin inhibitory proteins, Nogo-A, myelin-associated glycoprotein (MAG), and oligodendrocyte–myelin glycoprotein (OMgp), have been reported to serve as ligands for NgR^22,23^. Although Lio et al. showed that substratum adherence and migration of human U87MG glioma cells were significantly attenuated by the expression of the extracellular domains of Nogo-66 and myelin-associated glycoprotein (MAG)^24^, the molecular mechanisms are still unclear. Furthermore, the functional role of NgR maturation in the migration and invasion of GBM remains largely unknown.

Thus, in this study, we analyzed both the precursor and mature forms of NgR in GBM cells and investigated their role and mechanism in migration/invasive activity. Interestingly, we found that the vimentin–NgR interaction suppressed the maturation of NgR, which regulates the migration/invasive activity of GBM cells. To our knowledge, our study is the first to report a novel mechanism for the vimentin–NgR interaction in the invasiveness of brain cancer. These results might provide new insight into a potential therapeutic target for this disease.

## Materials and methods

### Cell line and culture conditions

The glioblastoma cell lines U87 and U251 were purchased from the European Collection of Authenticated Cell Cultures (Sigma-Aldrich Korea) and were maintained in DMEM supplemented with 100 U/ml penicillin, 100 µg/ml streptomycin, 25 ng/ml amphotericin B, and 10% fetal bovine serum (FBS) (GIBCO) at 37 °C in a humidified incubator with 5% CO_2_.

### Reagents and antibodies

The TβRI inhibitor LY2109761 (Cayman Chemical) and TGF-β1 (R&D systems) were used at the indicated concentrations. Specific antibodies against TGFβ1 (Cell Signaling), Id1 (B-8; Santa Cruz Biotechnology), Nogo Receptor (Abcam), E-cadherin, N-cadherin (BD Biosciences), and β-actin (Sigma-Aldrich) were used for immunoblotting. Antibodies against IgG (Cell Signaling) and Nogo Receptor were used for FACS analysis and immunoprecipitation with Pierce protein A/G Agarose (Thermo Scientific). Matrigel Basement Membrane Matrix (Corning) and Recombinant Human OMgp (R&D systems) were used for the cell-matrix adhesion assay and transwell migration/invasion assay.

### SDS–PAGE, in-gel digestion, and LC–MS/MS analysis

Protein samples (30 µg) were separated by 12% SDS–PAGE, followed by staining with Coomassie Brilliant Blue R-250. The SDS–PAGE gel was cut into two pieces based on the molecular weight (one piece included a 55-kDa protein, and the other included a 40-kDa protein). Sliced gels were digested with trypsin (Promega) for 16 h at 37 °C after reduction with 10 mM dithiothreitol (DTT) and the alkylation of cysteines with 55 mM iodoacetamide. The digested peptides were recovered with the extraction solution (50 mmol/L ammonium bicarbonate, 50% acetonitrile, and 5% trifluoroacetic acid). The extracted tryptic peptides were dissolved in 0.5% trifluoroacetic acid prior to further fractionation by LC–MS/MS analysis.

### LC–MS/MS analysis

Tryptic peptide samples were loaded onto a 2G-V/V trap column (Waters) for the enrichment of peptides and the removal of chemical contaminants. Concentrated tryptic peptides were eluted from the column and directed onto a 10-cm × 75-µm i.d. C18 reverse-phase column (PROXEON) at a flow rate of 300 nl/min. Peptides were eluted by using a gradient of 0–65% acetonitrile for 80 min. All MS and MS/MS spectra were acquired in a data-dependent mode by using an LTQ-Velos ESI Ion Trap mass spectrometer (Thermo Scientific). Each full MS (m/z range of 300–2000) scanned the most abundant precursor ions in the MS spectra. For protein identification, MS/MS spectra were analyzed by MASCOT (Matrix Science, http://www.matrixscience.com). The protein sequence database entry for RTN4R (CAK54501.1) was downloaded from NCBI and was used for the protein identification of precursor and mature Nogo receptors. The mass tolerance of the parent or fragment ion was 0.8 Da. The carbamidomethylation of cysteine and the oxidation of methionine were considered to be variable modifications of tryptic peptides in the MS/MS analysis.

### RT-PCR and real-time PCR

Oligonucleotide sequences corresponding to the vimentin, SLUG, and snail genes were designed by using Primer3 software (http://frodo.wi.mit.edu). The first-strand cDNA mixture contained 0.5 μg of total RNA as a template for RT-PCR and real-time PCR. TOPsimple PreMIX (2×; Enzynomics) was used to perform RT-PCR, and BioFACT^TM^ 2X Real-time PCR Master Mix (Biofact Biofactory) was used to perform real-time PCR according to the manufacturer’s protocol. The primer sequences for GAPDH were 5′-GGTATCGTGGAAGGACTC-3′ (sense) and 5′-GTAGAGGCAGGGATGATG-3′ (antisense). The vimentin-specific primers used for PCR were as follows: 5′-AATGGCTCGTCACCTTCGTGAAT-3′ (sense) and 5′-CAGATTAGTTTCCCTCAGGTTCAG-3′ (antisense). The SLUG-specific primers were as follows: 5′-GAGTCTGTAATAGGATTTCCCATAG-3′ (sense) and 5′-CTTTAGTTCAACAATGGCAAC-3′ (antisense). The Snail-specific primers were as follows: 5′-TTGGATACAGCTGCTTTGAG-3′ (sense) and 5′-ATTGCATAGTTAGTCACACCTC-3′ (antisense). Optimized real-time PCR was carried out as follows: one cycle of 95 °C for 15 min; 40 cycles of 95 °C for 20 s, and 60 °C for 40 s. The relative gene expression levels were normalized to GAPDH expression.

### Western blot analysis

Sodium dodecyl sulfate–polyacrylamide gel electrophoresis (SDS–PAGE) was conducted by using a Mini-PROTEIN® System (Bio-Rad, USA) and a 12% gel according to the manufacturer’s protocol. Proteins were transferred to a nitrocellulose blotting membrane and were probed with primary antibodies, followed by an HRP-conjugated secondary antibody. Immunolabeled proteins were detected by incubation with an enhanced chemiluminescence (ECL) substrate, followed by the exposure of the membrane to autoradiography film.

### WST-1 assay and FACS analysis

U87 and U251 were treated with 20 μM LY2109761 or 2 ng/ml TGFβ1 for 72 h and were subjected to survival analysis by using the WST-1 assay (Boehringer Mannheim) according to the manufacturer’s protocol. To determine the surface NgR expression and the intracellular expression of NgR and vimentin after treatment with LY2109761 or TGFβ1, FACS analysis was conducted with a guava easyCyte Systems and InCyte 3.1 software (Merck Millipore, Bedford, MA).

### Scratch–wound migration assay

U87 and U251 were treated with 20 μM LY2109761 or 2 ng/ml TGFβ1 for 48 h before the assay, and wounds were created by scratching using a 200-μl pipette tip. Duplicate wells were set up for each condition, and three fields per well were captured at each time point over a period of 48 h.

### Cell-matrix adhesion assay

A 96-well microtiter plate was coated with Matrigel (50 μl/well) for 1 h at 37 °C and was then blocked with BSA (10 mg/ml). After being treated with OMgp (100 ng/ml) for 2 h at 37 °C, U87 and U251 cells were then seeded onto these components 48 h after treatment with LY2109761 or TGFβ1. The cells were allowed to adhere to each well for 2 h at 37 °C and were gently washed three times with PBS. The adhesion of U87 and U251 cells to the extracellular components was quantified by counting three random fields per well under a microscope. All experiments were performed in triplicate.

### Cell migration assays

A transwell migration assay was conducted by using BD Falcon^TM^ Cell Culture Inserts. U87 and U251 cells were treated with LY2109761 or TGFβ1 for 48 h before the assay. The upper chamber of the transwells was seeded with 0.2 ml of cells (4 × 10^5^ cells/ml) in media with 5% FBS with a half of the treatment only or with the treatment and OMgp (100 ng/ml), and 0.6 ml of DMEM containing 20% FBS was added to the lower chambers. The cells were incubated in the transwells at 37 °C in 5% CO_2_ for 24 h. Cells that had migrated were stained with crystal violet. The migrated cells in each well were counted under a microscope in three fields per experiment. The mean values were obtained from three replicate experiments and were subjected to a *t* test.

### Cell invasion assays

U87 and U251 cells were treated with LY2109761 or TGFβ1 for 48 h before the assay. The cells were harvested by trypsinization and were washed in serum-free DMEM containing soybean trypsin inhibitor (2 mg/ml). The cells were suspended in serum-free medium at 4 × 10^5^ cells/ml. Prior to preparing the suspended cells, a dried layer of Matrigel (100 μl/well) with OMgp (100 ng/ml) or Matrigel matrix only was rehydrated with serum-free DMEM medium for 2 h at 37 °C. The rehydration solution was carefully removed, 0.1 ml of culture medium with a half of the treatment was added to the upper chambers, and 0.1 ml (4 × 10^4^ cells) of cell suspension was added to each lower chamber (with 5% FBS). The lower chambers were treated with 0.6 ml of DMEM containing 20% FBS. The plates were incubated for 24 h at 37 °C. Cells that had invaded the bottom surface of the membrane were stained with crystal violet. The cells were counted by taking photomicrographs at ×100 magnification. Cells in three different fields of each well were counted with two wells per treatment. The mean values were obtained from three replicate experiments and were subjected to a *t* test.

### Laser-scanning confocal microscope analysis

U87 and U251 cells were treated with LY2109761 or TGFβ1 for 48 h before confocal microscopy analysis. Then, the cells were fixed in 4% paraformaldehyde in 0.1 M PB (pH 7.4) at 4 °C overnight. All the samples were blocked with 5% goat serum in 0.2% Triton X-100 for 1 h at room temperature (RT) and were then incubated overnight at 4 °C with anti-TGFβ (1:500), E-cadherin (1:500), NgR (1:500), Id1 (1:1000), vimentin (1:1000), and β-catenin (1:1000) antibodies. The subsequent procedures were previously described^25^.

### Immunoprecipitation analysis

Cell lysates were incubated with a Nogo receptor antibody or control IgG overnight at 4 °C, and antigen–antibody complexes were precipitated with Pierce protein A/G Agarose (Thermo Scientific) for 2 h at room temperature. The immunoprecipitated complexes were cleared and analyzed by Western blotting as described above.

### Small-interfering RNA transfection

Vimentin small interfering RNA (siRNA) and control siRNA were purchased from Bioneer Co. (Daejeon, Korea). The primer sequences of vimentin siRNA #1 were sense 5′-UGA AGC UGC UAA CUA CCA ATT-3′ and antisense 5′-UUG GUA GUU AGC AGC UUC ATT-3′. The primer sequences of vimentin siRNA #2 were sense 5′-UCA CCU UCG UGA AUA CCA ATT-3′ and antisense 5’-UUG GUA UUC ACG AAG GUG ATT-3′. U87 and U251 cells were transfected with vimentin siRNA or control siRNA by using Lipofectamine Plus (Invitrogen) according to the manufacturer’s protocol.

### Lentivirus infections

Plasmids containing shRNAs for human vimentin (TRCN0000029119, TRCN0000029120, TRCN0000297192, and TRCN0000297191, Sigma) or a scrambled shRNA (#1864, Addgene, Cambridge, MA) were cotransfected with pVSV-G and a packaging plasmid (SBI, Palo Alto, CA) into HEK293T cells by using the Lipofectamine 3000 transfection reagent (Thermo Scientific, Waltham, MA). TRCN0000029119, TRCN0000029120, TRCN0000297192, and TRCN0000297191 were designated shVIM1, shVIM2, shVIM3, and shVIM4, respectively. GBM cell lines were incubated with viral supernatants from HEK293T cells and polybrene (5 μg/ml) for 48 h. After 10 days of selection with puromycin (1.5 μg/ml), the efficiency of vimentin knockdown was evaluated by Western blotting.

### Overall survival analysis by using TCGA data

The RNA-seq data and clinical information from low-grade glioma patients from The Cancer Genome Atlas (TCGA) project were downloaded from the data portal of International Cancer Genome Consortium (ICGC) (release 25) (https://dcc.icgc.org/). We divided the patients into two or four groups according to their normalized read counts of the *VIM* and *RTN4R* genes and then performed survival analysis. All statistical tests were performed by using the R programming language (https://www.r-project.org/), and the graphs were prepared by using R.

### Statistical analysis

Data are shown as the mean ± the standard deviation, and the significance of the statistical analysis was assessed by using a two-tailed, unpaired Student’s *t* test. The level of statistical significance stated in thispaper is based on the *p* values. *p < 0.01, **p < 0.005, or ***p < 0.001 was considered statistically significant.

## Results

### Expression of precursor and mature NgR in glioblastoma cell lines

Many previous studies have shown that TGFβ1 induces the invasion of GBM. A previous paper indicated that NgR inhibits the migration and invasion of human glioma cells^24^. Since the maturation process of NgR is essential for the interaction of NgR with other ligands, we hypothesized that TGFβ1 might be associated with the conversion of the NgR isoform.

**Fig. 1:**
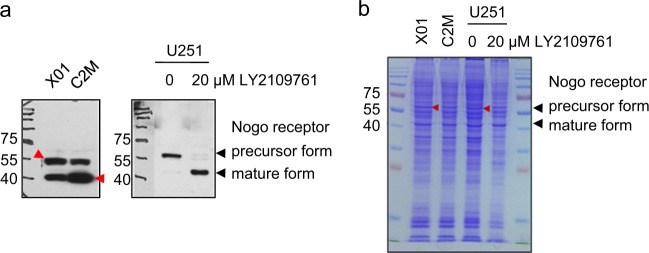
Analysis of precursor and mature NgR in GBM cells. **a** Western blot analysis showing the precursor and mature form of Nogo receptor in X01, C2M, and in untreated or 20 μM LY2109761-treated U251 cells. **b** Coomassie staining of a 12% SDS–PAGE gel with CBB R-250. In-gel digestion was conducted for LC–MS/MS analysis, as described in the Supplementary data (available online, red arrow)

The Western blot analysis of NgR in GBM and GBM stem-like cells (GBMSCs) showed two different isoforms of NgR (Supplementary Fig. 1a). Based on UniProt data (https://www.uniprot.org/uniprot/Q9BZR6#expression), NgR is a 473-amino acid protein (52 kDa), and the removal of 26 amino acids on the C terminus results in a 49-kDa mature form. Two GBMSC cell lines (X01 and C2M) expressed both isoforms, but a GBM cell line (U251) mainly expressed the precursor form. Intriguingly, in U251 cells, the TGFβ receptor type I inhibitor LY2109761 induced the conversion of the precursor to the mature isoform of NgR (Fig. 1a). To the best of our knowledge, there have been no reports on the isoform of NgR. Therefore, we tried to validate whether the two bands from Western blotting analysis were isoforms of NgR. From SDS–PAGE gels of X01, C2M, and U251 cell lysates, we isolated four bands at the predicted size of the precursor and mature forms of NgR, and their sequences were analyzed by LC–MS/MS analysis (Fig. 1b). The precursor form in X01 and LY2109761-untreated U251 cells (Supplementary Fig. 2a and c) and the mature form in C2M and LY2109761-treated U251 cells (Supplementary Fig. 2b and d) were confirmed. Our results indicate that two different isoforms of NgR are expressed in GBM cells, and the maturation of NgR could be affected by signaling pathways, including the TGFβ pathway.

### Maturation of NgR is associated with the migration of GBM cells

**Fig. 2:**
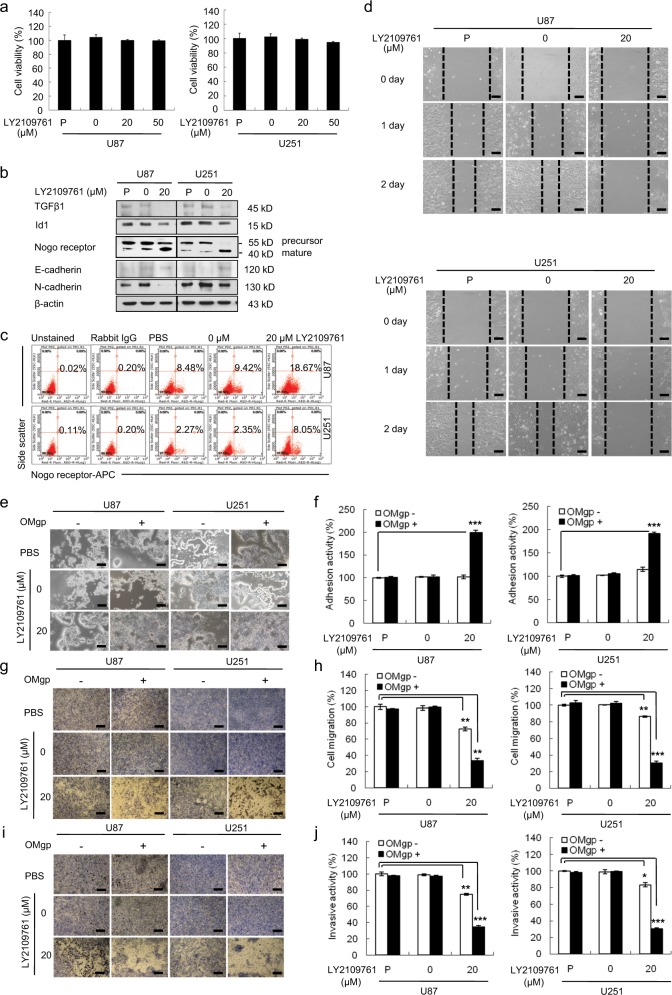
Role of mature NgR on cellular behavior in LY2109761-treated GBM. **a** WST-1 assay revealed that the cell viability was not affected by LY2109761 treatment in U87 and U251 cells. **b** Western blot analysis showing the expression levels of TGFβ1, Nogo receptor, and E-cadherin in LY2109761-treated U87 and U251 cells. The expression levels of all proteins were normalized to those of β-actin. **c** FACS analysis showing that the inhibition of TGFβ1 resulted in increases in mature surface Nogo receptor. **d** The scratch–wound migration activity of LY2109761-treated U87 and U251 cells, as determined by the scratch–wound migration assay. U87 and U251 cells were treated with 20 μM LY2109761 for 48 h prior to the assay, and the wound was created by scratching using a sterile 200-μl pipette tip. Duplicate wells were used for each condition, and three fields per well were captured at each time point over a period of 48 h. Images of the same fields were taken at days 0–2 (×100 magnification). Black scale bar = 100 μm. The inhibition of TGFβ1 suppressed the migratory ability of U87 and U251 cells. **e** The adhesion activity of LY2109761-treated U87 and U251 cells, as determined by an OMgp-coated matrix adhesion assay. Phase-contrast image of U87 and U251 cells treated with LY2109761, showing representative cells adhering to the well with or without OMgp (100 ng/ml) (×100 magnification). Black scale bar = 100 μm. **f** Quantification of adhesive U87 and U251 cells treated with LY2109761. The inhibition of TGFβ1 increased the adhesion activity of U87 and U251 cells through the upregulation of OMgp responsiveness. **g** Cell migration activity of LY2109761-treated U87 and U251 cells, as determined by an OMgp-treated transwell migration assay. U87 and U251 cells were treated with LY2109761 for 48 h before the assay. The upper chamber of the transwells was seeded with 0.2 ml of cells (4 × 10^5^ cells/ml) in medium with 5% FBS supplemented with half the amount of the treatment only or with OMgp (100 ng/ml), and 0.6 ml of DMEM containing 20% FBS was added to the lower chambers. After 24 h, migrating cells were stained with crystal violet, and images were taken (×100 magnification). Black scale bar = 100 μm. **h** The migrating cells were counted under a microscope in three different fields per experiment. **i** Cell invasion was examined through a membrane filter coated with OMgp/Matrigel or Matrigel alone. After 24 h, invading cells were stained with crystal violet, and images were taken (×100 magnification). Black scale bar = 100 μm. **j** The invading cells were counted under a microscope in three different fields per experiment. The mean values and the standard error were obtained from three individual experiments. *p < 0.01, **p < 0.005, and ***p < 0.001

To investigate the functional role of the increased expression of mature NgR in GBM cells, we induced the maturation of NgR by inhibiting TGFβ1 with LY2109761 in these cells, and the functional changes were investigated (Fig. 2). While there was no difference in the cell viability of U87 or U251 cells regardless of LY2109761 treatment (Fig. 2a), the inhibition of TGFβ1 suppressed the expression of N-cadherin and induced mature NgR and E-cadherin expression (Fig. 2b). Furthermore, LY2109761 (20 μM) treatment significantly increased the expression of mature cell-surface NgR to 18.67 and 8.05% in U87 and U251 cells, respectively, compared with 8.48 and 2.27% in untreated U87 and U251 cells (Fig. 2c). Thus, these results demonstrate that TGFβ1 inhibition by LY2109761 leads to the induction of NgR maturation.

We then assessed whether the induction of mature NgR affects the migration of U87 and U251 cells. A scratch–wound migration assay was performed by using cells treated with LY2109761. As shown in Fig. 2d, no movement was observed in cells treated with 20 μM of LY2109761, while PBS-treated cells showed significant movement. To confirm whether migration was mediated by the maturation of NgR, inhibitors of carboxypeptidase Y (CpY), Z–L-phechloromethylketone (ZPCK), and aprotinin were used in the scratch–wound migration assay. These drugs block the digestion of the 447S of NgR polypeptides, resulting in the suppression of NgR maturation. While cell viability was not significantly affected, migration obviously increased as the results of the inhibition of CpY (Supplementary Fig. 3), suggesting that NgR maturation in GBM was associated with migration. Overall, these results provide evidence that the inhibition of TGFβ1 induced the expression of surface NgR and significantly suppressed the migration ability of U87 and U251 cells.

### Induction of surface NgR expression enhances cell adhesion and suppresses migration and invasion through the upregulation of OMgp responsiveness

Next, we performed a modified cell-matrix adhesion assay by using OMgp-coated plates to determine whether the upregulated surface NgR induced by LY2109761 interacts with a ligand. Two hours after seeding cells on OMgp-coated plates, the number of viable adherent cells had significantly increased to 199.8 or 191.7% in LY2109761-treated U87 and U251 cells, respectively, compared with that in control-treated cells (Fig. 2e, f). These results indicate that the upregulated surface NgR increases the cell adhesion of GBM cells to OMgp-coated Matrigel but does not affect the cell adhesion to OMgp-uncoated Matrigel.

Next, we examined whether the upregulation of OMgp responsiveness in GBM cells treated with LY2109761 also affected the migration and invasion abilities of U87 and U251 cells (Fig. 2g, i). As shown in Fig. 2h, 20 μM LY2109761 significantly reduced the percentage of migrating cells to 33.2 and 30.4% in U87 and U251 cells, respectively, compared with untreated U87 (97%) and U251 (99.7%) cells. However, in the migration chamber without OMgp, the percentage of migrating cells in LY2109761-treated U87 or U251 cells decreased to 72.3 or 86.3%, respectively, compared with 100% in untreated cells. Consistently, LY2109761 suppressed the percentage of invasive U87 and U251 cells to 65.3 or 69.7%, respectively, compared with untreated cells (100%), as demonstrated by the Matrigel with OMgp-coated invasion assay (Fig. 2j). However, in the Matrigel without OMgp-coated invasion chamber, the percentage of invasive U87 and U251 cells after treatment with LY2109761 decreased to 25.4 and 16.6%, respectively, compared with that in untreated control cells (Fig. 2j). Overall, the enhanced OMgp responsiveness of U87 or U251 cells after treatment with LY2109761 caused a 2–4-fold decrease in cell migration and invasive activity compared with cells cultured without OMgp. These results suggest that the induction of mature NgR in GBM cells suppresses OMgp-coated transwell migration and invasive activity by enhancing OMgp responsiveness.

### Increase in TGFβ1 levels by recombinant human TGFβ1 enhances the migration and invasion of U87 and U251 cells

**Fig. 3:**
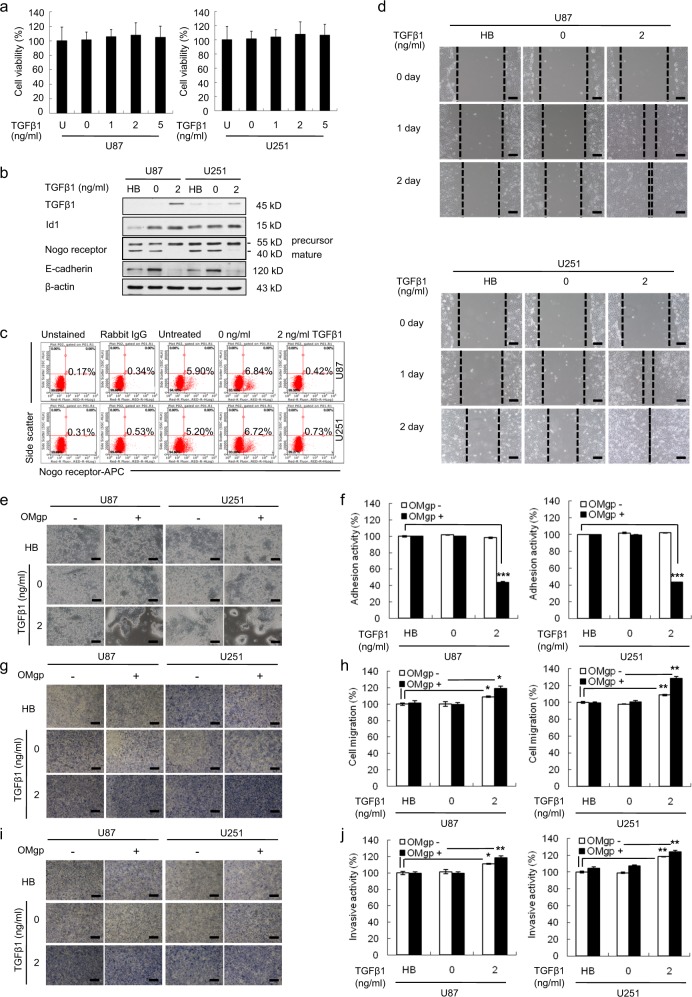
In vitro effects of TGFβ1 treatment in U87 and U251 cells. **a** WST-1 assay revealed that cell viability was not affected by TGFβ1 treatment in U87 and U251 cells. **b** Western blot analysis showing the expression levels of TGFβ1, Nogo receptor, and E-cadherin in U87 and U251 cells treated with TGFβ1. The expression levels of all proteins were normalized to those of β-actin. **c** FACS analysis showing that the activation of TGFβ1 suppresses mature cell-surface Nogo receptor. **d** The scratch–wound migration activity of U87 and U251 cells treated with TGFβ1 was enhanced. U87 and U251 cells were treated with 2 ng/ml TGFβ1 for 48 h prior to the assay, and the wound was scratched by using a 200-μl sterile pipette tip. Duplicate wells were used for each condition, and three fields per well were captured at each time point over a period of 48 h. Images of the same fields were taken at days 0–2 (×100 magnification). Black scale bar = 100 μm. **e** Phase-contrast image of U87 and U251 cells after treatment with TGFβ1, showing representative cells adhering to the well with or without OMgp (100 ng/ml) (×100 magnification). Black scale bar = 100 μm. **f** Quantification of adhesive U87 and U251 cells after treatment with TGFβ1. Treatment with TGFβ1 decreased the adhesion activity of U87 and U251 cells through the suppression of OMgp responsiveness. **g** Cell migration activity of U87 and U251 cells treated with TGFβ1, as determined by an OMgp-treated transwell migration assay. U87 and U251 cells were treated with TGFβ1 for 48 h before the assay. The upper chamber of the transwells was seeded with 0.2 ml of cells (4 × 10^5^ cells/ml) in medium with 5% FBS supplemented with half of the drug treatment only or half of the drug treatment with OMgp (100 ng/ml), and 0.6 ml of DMEM containing 20% FBS was added to the lower chambers. After 24 h, migrating cells were stained with crystal violet, and images were taken (×100 magnification). Black scale bar = 100 μm. **h** The migrating cells were counted under a microscope in three different fields per experiment. **i** Cell invasion was examined through a membrane filter coated with OMgp/Matrigel or Matrigel alone. After 24 h, invading cells were stained with crystal violet, and images were taken (×100 magnification). Black scale bar = 100 μm. **j** The invading cells were counted under a microscope in three different fields per experiment. The mean values and the standard error were obtained from three individual experiments. *p < 0.01, **p < 0.005, and ***p < 0.001

To evaluate the effect of TGFβ1 on GBM cells, we performed cell proliferation, flow cytometry, scratch–wound migration, cell-matrix adhesion, transwell migration, and invasion assays (Fig. 3). Treatment with recombinant human TGFβ1 did not change the viability of U87 and U251 cells compared with untreated cells (Fig. 3a). As anticipated, the addition of recombinant human TGFβ1 reduced the expression of mature NgR and E-cadherin (Fig. 3b). Furthermore, mature cell-surface NgR expression decreased in TGFβ1 (2 ng/ml)-treated U87 (0.42%) and U251 (0.73%) cells compared with control U87 (6.84%) and U251 (6.72%) cells (Fig. 3c). As shown in Fig. 3d, the width of the wound was significantly narrower after 48 h of TGFβ1 treatment in U87 and U251 cells compared with that in untreated control cells.

In addition, the suppression of surface NgR by treatment with TGFβ1 significantly inhibited adhesion to OMgp-coated Matrigel in U87 (43.6%) and U251 (43.1%) cells, respectively, compared with untreated U87 and U251 cells (100%) (Fig. 3e, f). Furthermore, in the migration chamber containing OMgp, the percentage of migrating cells in TGFβ1-treated U87 and U251 cells was significantly higher (119.2 and 128.5%, respectively) than that of replicates in untreated U87 (101.4 and 99.5%) or U251 (99.7 and 100.7%) cells (Fig. 3g, h). However, in the migration chamber without OMgp, the migration activity of TGFβ1 (2 ng/ml)-treated U87 and U251 cells increased to 109 and 108%, respectively, compared with the two control groups of U87 (100 and 100.4%) and U251 (100 and 98%) cells, respectively (Fig. 3g, h). In line with this observation, in the Matrigel with OMgp-coated invasion chamber, the invasive ability of TGFβ1 (2 ng/ml)-treated U87 and U251 cells increased up to 118.0 and 124.4% compared with that of the two control groups of U87 (99.6 and 99.7%) and U251 (104.7 and 99.1%) cells, respectively (Fig. 3i, j). In the Matrigel without OMgp-coated invasion chamber, the percentage of invading cells in TGFβ1-treated U87 and U251 cells was higher (111 and 118.4%, respectively) than that in the two untreated U87 (100 and 101.4%) and U251 (100 and 99.1%) cells (Fig. 3i, j).

Collectively, these results provide evidence that the upregulation of TGFβ1 enhances the migration and invasion of U87 and U251 cells by suppressing the mature surface NgR, resulting in the downregulation of OMgp responsiveness.

### The vimentin–NgR complex plays a crucial role in the TGFβ1-mediated migration and invasion of GBM cells

**Fig. 4:**
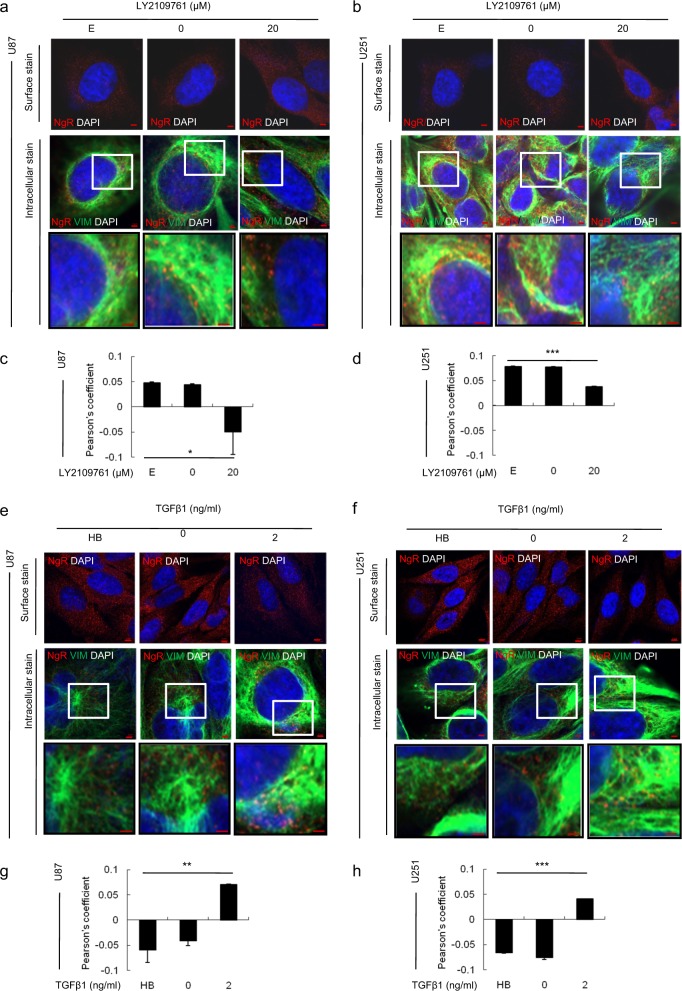
The cellular localization of the Nogo receptor and vimentin proteins in GBM cells treated with LY2109761 or TGFβ1 by using a laser-scanning confocal microscope. **a**, **b**, **e**, **f** Immunofluorescence staining demonstrated that TGFβ1 inhibition increased Nogo receptor expression in U87 and U251 cells, and that the activation of TGFβ1 suppresses the expression of Nogo receptor (panel of surface stains). Intracellular staining shows that the cellular colocalization of Nogo receptor and vimentin decreased in U87 and U251 cells treated with LY2109761, while this colocalization increased in TGFβ1-treated U87 and U251 cells. Red scale bar = 50 μm. Inset images are zoomed twofold. **c**, **d**, **g**, **h** Quantification of the colocalization of Nogo receptor and vimentin. Pearson’s coefficient was automatically calculated from two randomly taken photographs in the field with low magnification. A representative image is presented in Supplementary Fig. 4e. The values are presented as the mean ± SD from three independent experiments. *p < 0.01, **p < 0.005, and ***p < 0.001

To elucidate the molecular mechanism of NgR in the migration and invasion of GBM cells through the TGFβ1 signaling pathway, the expression and localization of several genes related to the regulation of NgR as well as the EMT signaling cascade were examined by confocal microscopy (Supplementary Fig. 4 and Fig. 4). Importantly, we observed a decrease in the colocalization of intracellular NgR and vimentin in U87 and U251 cells after treatment with LY2109761 (Fig. 4a–d). On the other hand, as expected, an increase in the colocalization of NgR and vimentin was observed in the cytoplasm of U87 and U251 cells treated with TGFβ1 (Fig. 4e–h). In addition, FACS analysis of intracellular NgR and vimentin staining also showed the same results (Supplementary Fig. 5) as confocal microscopy. Together, these results indicate that the inhibition of TGFβ1 by LY2109761 enhances the surface expression of NgR via the vimentin-mediated maturation of NgR.

**Fig. 5:**
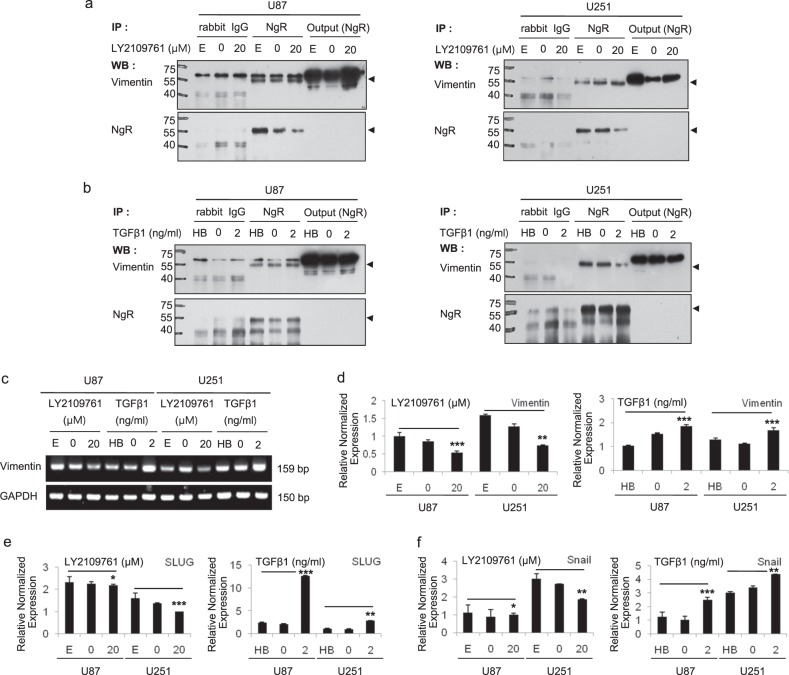
The interaction between intracellular NgR and vimentin in GBM cells. **a** Immunoprecipitation by using the NgR antibody showed that the size of precursor Nogo receptor was decreased upon the inactivation of the TGFβ1 pathway by LY2109761 in NgR antibody-immunoprecipitated U87 and U251 total cell lysates, and vimentin expression was similar between these samples. **b** Nogo receptor was not different in response to the activation of the TGFβ pathway in U87 and U251 cells. **c**, **d** The transcriptional level of vimentin was significantly downregulated in LY2109761-treated U87 and U251 cells compared with control cells based on RT-PCR and real-time PCR analysis. **e**, **f** Two upstream genes of the EMT signaling cascade, which includes Vimentin, Slug, and Snail, were transcriptionally suppressed in U87 and U251 cells by the inhibition of TGFβ1, while these genes were transcriptionally upregulated compared with control cells in these cells upon treatment with TGFβ1. All detected genes were normalized to *GAPDH*. The mean values and the standard error were obtained from three individual experiments. *p < 0.5, **p < 0.1, and ***p < 0.05

In support of the observation that NgR interacts with vimentin, we performed an immunoprecipitation assay by using an NgR antibody (Fig. 5). In both the LY2109761 and TGFβ1 treatments, we found that vimentin coprecipitated with NgR, which is consistent with the colocalization results shown in Fig. 4. Interestingly, as shown in Fig. 5a, the expression of the precursor form of NgR decreased upon the inactivation of the TGFβ1 pathway by LY2109761 treatment, indicating that LY2109761 increased the mature form of NgR. These results show that vimentin interacts with NgR, and this complex plays a key role in GBM migration and invasion through the regulation of NgR maturation and surface expression via the TGFβ1 pathway.

To further study the involvement of EMT in GBM cells treated with LY2109761 or TGFβ1, we examined the expression of vimentin, Slug, and Snail. RT-PCR and qPCR analysis (Fig. 5c, d) revealed that the transcriptional level of vimentin was significantly downregulated in LY2109761-treated U87 and U251 cells compared with untreated cells. Moreover, we observed that treatment with TGFβ1 significantly upregulated the expression of vimentin, Slug, and Snail in U87 and U251 cells (Fig. 5e, f). Thus, these results suggest that the EMT signaling cascade is involved in the migration and invasion of GBM cells treated with TGFβ1.

### Knockdown of vimentin suppressed migration activity through the maturation of NgR

**Fig. 6:**
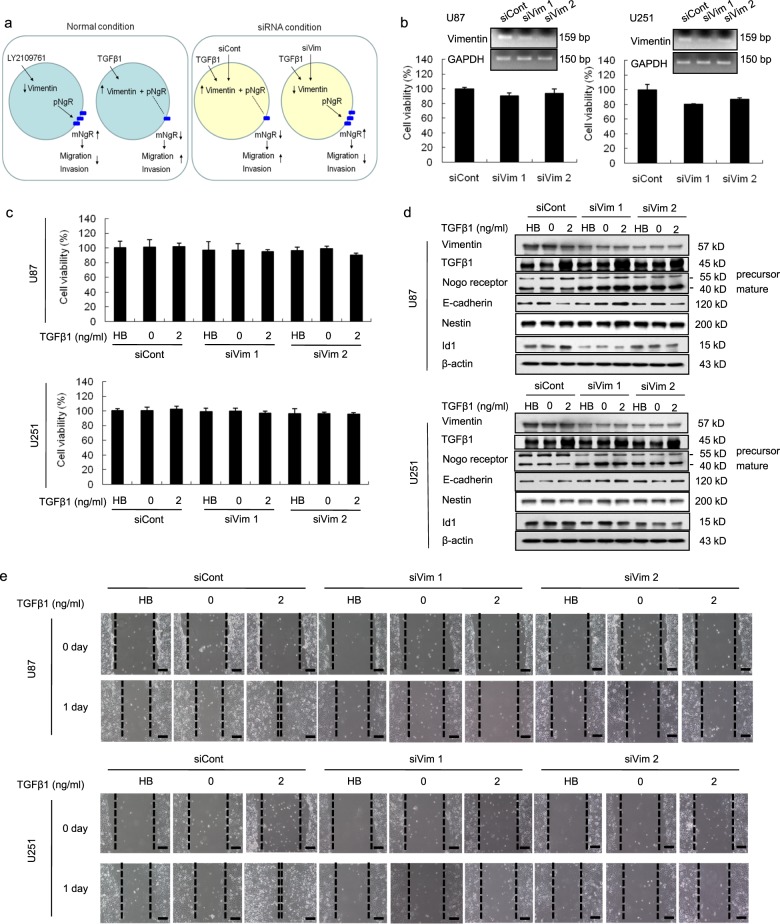
Knockdown of vimentin attenuated the migration of TGFβ1-treated U87 and U251 cells. **a** A hypothetical mechanism showing that migration activity is decreased in vimentin siRNA-transfected GBM cells that are treated with TGFβ1. **b** WST-1 assay and the confirmation of vimentin silencing by RT-PCR. U87 and U251 cells were transfected with vimentin siRNA or control siRNA constructs for 1 day, were treated with TGFβ1 (2 ng/ml) for 2 days, and were subjected to the following assays. **c** Proliferation activity of U87 and U251 cells was measured by a WST-1 assay. **d** Western blot analysis showing the expression levels of vimentin, TGFβ1, Nogo receptor, E-cadherin, and Nestin in vimentin-silenced, TGFβ1-treated U87 and U251 cells. The expression levels of all proteins were normalized to those of β-actin. **e** The scratch–wound migration activity of the vimentin-silenced, TGFβ1-treated U87 and U251 cells was suppressed, while the activity was enhanced in control siRNA-transfected U87 and U251 cells. Duplicate wells were used for each condition, and three fields per well were captured at each time point over a period of 24 h. Images of the same fields were taken at days 0 and 1 (×100 magnification). Black scale bar = 100 μm

Based on our previous results, we hypothesized that the suppression of vimentin expression would inhibit the migration and invasion of GBM cells through the maturation of NgR (Fig. 6a). To verify this hypothesis, we analyzed cell proliferation, the related protein expression, and scratch–wound migration assays with transient or permanent silencing of vimentin in U87 and U251 cells (Fig. 6 and Supplementary Fig. 6). There was no difference in the viability of vimentin-silenced U87 or U251 cells (Fig. 6b, c) regardless of treatment with TGFβ1. While the activation of TGFβ1 suppressed the maturation of NgR during control siRNA treatment, we did not observe the suppression of the mature form of NgR expression in vimentin-silenced cells (Fig. 6d). Similar results were also found in cells that underwent permanent vimentin knockdown by using shRNA (Supplementary Fig. 6).

### Survival analysis for the association between NgR and vimentin

**Fig. 7:**
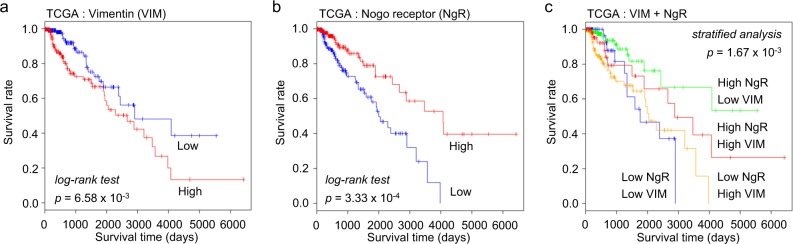
Overall survival analysis of glioma patients based on vimentin and Nogo receptor combinations. **a** Prognosis of two groups of glioma patients classified by vimentin expression (log-rank *p* = 6.58 × 10^**−**3^ for TCGA release 26) (*n* = 428). **b** Prognosis of two groups of glioma patients classified by Nogo receptor expression (log-rank *p* = 3.33 × 10^**−**4^ for TCGA release 26) (*n* = 428). **c** Prognosis of glioma patient groups stratified by vimentin and Nogo receptor expression (green: high NgR, low VIM; red: high NgR, high VIM; blue: low NgR, low VIM; yellow: low NgR, high VIM) (*p* = 1.67 × 10^**−**3^)

Finally, we investigated the association between the survival rate for glioma patients and vimentin and/or NgR gene expression by using survival data from TCGA (Fig. 7). Survival was significantly lower in patients with high-vimentin expression than in patients with low-vimentin expression (log-rank *p* = 6.58 × 10^−3^, Fig. 7a). For NgR, the survival rate was better in the group with high expression than in the group with low expression (log-rank *p* = 3.33 × 10^−4^, Fig. 7b). In the stratified analysis, the high-NgR/low-vimentin expression group showed a significantly higher survival rate than the high-NgR/high-vimentin expression group (*p* = 1.67 × 10^−3^, Fig. 7c). These results were consistent with our in vitro results.

**Fig. 8:**
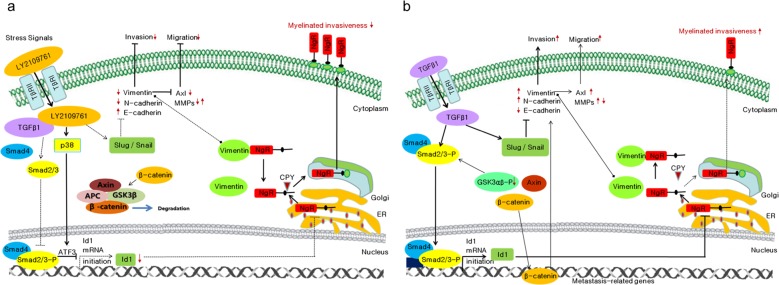
The proposed molecular mechanism showing that **a** TGFβ1 inhibition resulted in the suppression of migration and the invasion of GBM and **b** the activation of TGFβ1 enhances GBM migration and invasion

Thus, this study suggests that vimentin might act as another regulator of the Nogo receptor via the formation of a complex and may mediate the migration and invasion of TGFβ1-treated GBM cells. We hereby propose a molecular mechanism that shows that TGFβ1 inhibition results in the suppression of the migration and invasion of GBM cells (Fig. 8a) and vice versa (Fig. 8b).

## Discussion

GBM can aggressively infiltrate/invade normal brain tissues. The invasive nature of GBM makes it very difficult to treat patients with current therapies. A better understanding of the molecular biology of glioma invasion could allow the use of targeted therapy. One of the major routes of glioma dissemination is along white matter fiber tracts on myelin, which can lead to distant spread, for example, through the corpus callosum into the other hemisphere^26–28^. The migration/invasion of glioma cells is mediated by multiple factors, including ECM molecules, growth factors, and the activity of intracellular pathways that regulate cell motility^29^. In this study, we demonstrate a novel molecular mechanism for the migration/invasion of GBM through NgR–vimentin interactions. A previous study showed that Nogo-66 and MAG inhibit the adhesion and migration of NgR-expressing glioma cells^24^. The inhibition of cell migration and invasion kinetics might be caused by a complex mechanism with the ligands of NgR, OMgp, Nogo-A, and MAG. Since OMgp, a ligand for NgR, is an inhibitor of neurite outgrowth on myelin^30^, the surface expression of NgR is a critical factor that inhibits migration/invasion along white matter fiber tracts. Therefore, it is important to achieve a better understanding of the mechanisms involved in the regulation of NgR expression on the cell surface. To the best of our knowledge, this is the first study to demonstrate the function of the isoforms of NgR in GBM cells and to describe an underlying mechanism for the maturation of NgR.

This study was limited to cultured cells and clinical survival analysis. The survival analysis in Fig. 7 was restricted to IDH1-wild-type, low-grade glioma patients. As a result, the interaction between NgR and vimentin was confirmed to have clinical significance through clinical survival analysis. However, in the survival analysis of high-grade glioblastoma (*n* = 142, data not shown), the interaction between NgR and vimentin had no clinical significance. This result may be related to the mutation of several genes in high-grade glioblastoma^31^. Despite many novel findings in our study, more research is needed to confirm the role of NgR in vivo and to elucidate its underlying mechanism.

Our results showed that TGFβ1 appeared to be less effective than the inhibition of TGFβ1 with LY2109761, which might have been because TGFβ expression is constitutively very high in most GBM cells. Nevertheless, we provided clear evidence that the activation of the TGFβ1 pathway enhanced the myelinated invasiveness of GBM cells through the interaction of vimentin and NgR. In addition, we performed in vitro experiments by using LY2109761 in primary glioma stem cells (GBMSC), such as X01 and X03, as shown in Supplementary Fig. 7. The results of cell viability, Western blot, FACS, and scratch–wound migration assays after LY2109761 treatment in two primary GBMSCs were similar to those in U87 and U251. Based on these results, experiments are underway to examine the connection between NgR maturation and primary GBMSC infiltration or primary GBMSC stemness. Our findings expand upon the current understanding of the migration/invasion of GBM and imply that the maturation process of NgR via vimentin may be a therapeutic target for glioma.

## Supplementary information

Supplementary Figures and Legends
