# Supplementary material for: Nogo receptor–vimentin interaction: a novel mechanism for the invasive activity of glioblastoma multiforme
Source: Exp Mol Med. 2019 Oct 24;51(10):125. doi: 10.1038/s12276-019-0332-1 (PMC6813361; doi:10.1038/s12276-019-0332-1)
Supplement: Supplementary file 1 — Supplementary Figures and Legends [file 12276_2019_332_MOESM1_ESM.pdf]

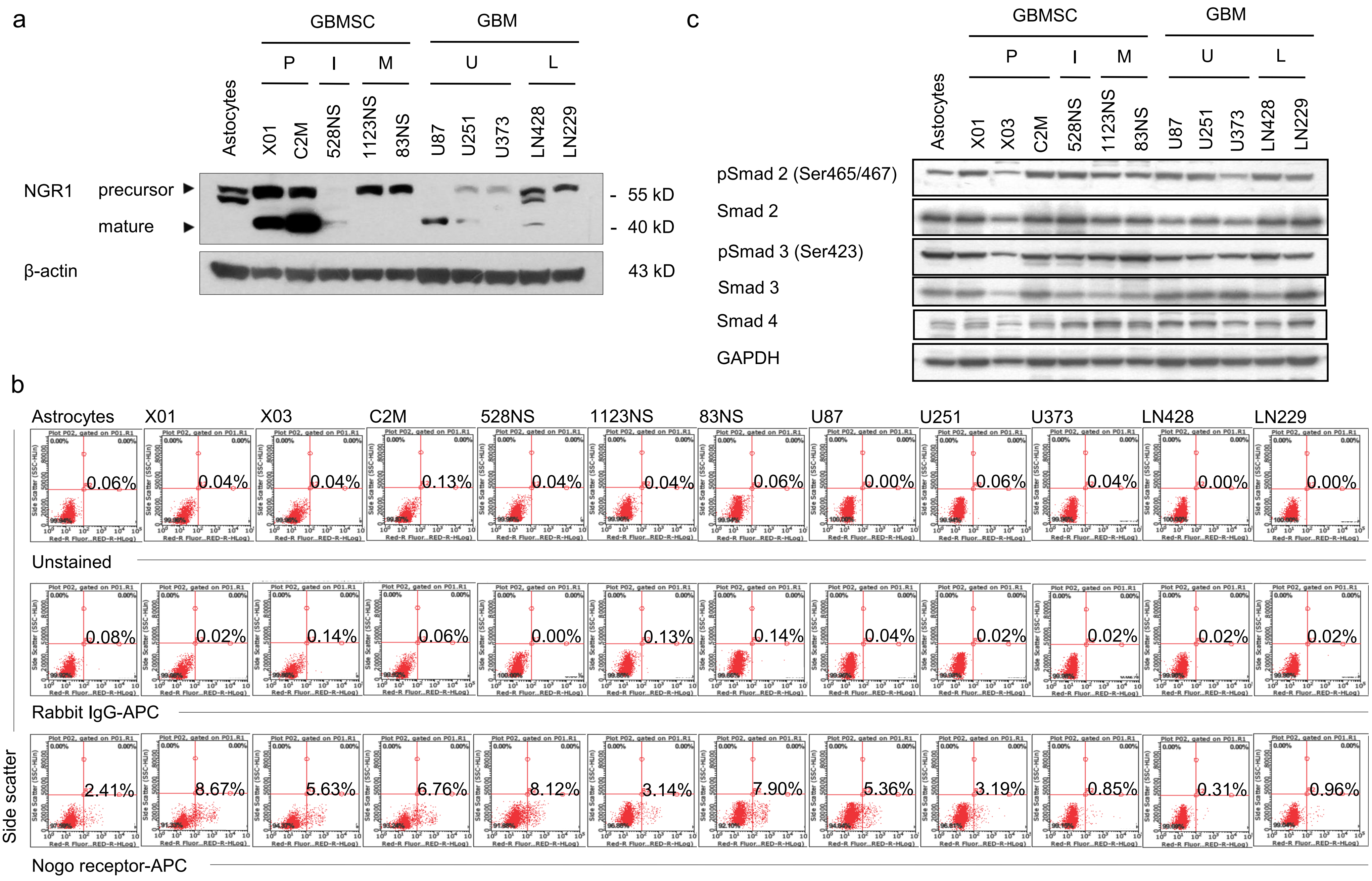

Supplementary Fig. 1

a

Protein sequence coverage: 87%

X01 precursor form

Matched peptides shown in ***bold red***.

```
  1 MKRASAGGSR LLAWVLWLQA WQVAAPCPGA CVCYNEPKVT TSCPQQGLQA
 51 VPVGIPAASQ RIFLHGNRIS HVPAASFRAC RNLTILWLHS NVLARIDAAA
101 FTGLALLEQL DLSDNAQLRS VDPATFHGLG RLHTLHLDRC GLQELGPGLF
151 RGLAALQYLY LQDNALQALP DDTFRDLGNL THLFLHGNRI SSVPERAFRG
201 LHSLDRLLLH QNRVAHVHPH AFRDLGRLMT LYLFANNLSA LPTEALAPLR
251 ALQYLRLNDN PWVCDCRARP LWAWLQKFRG SSSEVPCSLP QRLAGRDLKR
301 LAANDLQGCA VATGPYHPIW TGRATDEEPL GLPKCCQPDA ADKASVLEPG
351 RPASAGNALK GRVPPGDSPP GNGSGPRHIN DSPFGTLPGS AEPPLTAVRP
401 EGSEPPGFPT SGPRRRPGCS RKNRTRSHCR LGQAGSGGGG TGDSEGSGAL
451 PSLTCSLTPL GLALVLWTVL GPC
```

b

Protein sequence coverage: 45%

C2M mature form

Matched peptides shown in ***bold red***.

```
  1 MKRASAGGSR LLAWVLWLQA WQVAAPCPGA CVCYNEPKVT TSCPQQGLQA
 51 VPVGIPAASQ RIFLHGNRIS HVPAASFRAC RNLTILWLHS NVLARIDAAA
101 FTGLALLEQL DLSDNAQLRS VDPATFHGLG RLHTLHLDRC GLQELGPGLF
151 RGLAALQYLY LQDNALQALP DDTFRDLGNL THLFLHGNRI SSVPERAFRG
201 LHSLDRLLLH QNRVAHVHPH AFRDLGRLMT LYLFANNLSA LPTEALAPLR
251 ALQYLRLNDN PWVCDCRARP LWAWLQKFRG SSSEVPCSLP QRLAGRDLKR
301 LAANDLQGCA VATGPYHPIW TGRATDEEPL GLPKCCQPDA ADKASVLEPG
351 RPASAGNALK GRVPPGDSPP GNGSGPRHIN DSPFGTLPGS AEPPLTAVRP
401 EGSEPPGFPT SGPRRRPGCS RKNRTRSHCR LGQAGSGGGG TGDSEGSGAL
451 PSLTCSLTPL GLALVLWTVL GPC
```

c

Protein sequence coverage: 87%

U251 0 µM LY2109761 precursor form

Matched peptides shown in ***bold red***.

```
  1 MKRASAGGSR LLAWVLWLQA WQVAAPCPGA CVCYNEPKVT TSCPQQGLQA
 51 VPVGIPAASQ RIFLHGNRIS HVPAASFRAC RNLTILWLHS NVLARIDAAA
101 FTGLALLEQL DLSDNAQLRS VDPATFHGLG RLHTLHLDRC GLQELGPGLF
151 RGLAALQYLY LQDNALQALP DDTFRDLGNL THLFLHGNRI SSVPERAFRG
201 LHSLDRLLLH QNRVAHVHPH AFRDLGRLMT LYLFANNLSA LPTEALAPLR
251 ALQYLRLNDN PWVCDCRARP LWAWLQKFRG SSSEVPCSLP QRLAGRDLKR
301 LAANDLQGCA VATGPYHPIW TGRATDEEPL GLPKCCQPDA ADKASVLEPG
351 RPASAGNALK GRVPPGDSPP GNGSGPRHIN DSPFGTLPGS AEPPLTAVRP
401 EGSEPPGFPT SGPRRRPGCS RKNRTRSHCR LGQAGSGGGG TGDSEGSGAL
451 PSLTCSLTPL GLALVLWTVL GPC
```

d

U251 20 µM LY2109761 mature form

```
  1 MKRASAGGSR LLAWVLWLQA WQVAAPCPGA CVCYNEPKVT TSCPQQGLQA
 51 VPVGIPAASQ RIFLHGNRIS HVPAASFRAC RNLTILWLHS NVLARIDAAA
101 FTGLALLEQL DLSDNAQLRS VDPATFHGLG RLHTLHLDRC GLQELGPGL
151 FRGLAALQYLY LQDNALQALP DDTFRDLGNL THLFLHGNRI SSVPERAFRG
201 LHSLDRLLLH QNRVAHVHPH AFRDLGRLMT LYLFANNLSA LPTEALAPLR
251 ALQYLRLNDN PWVCDCRARP LWAWLQKFRG SSSEVPCSLP QRLAGRDLKR
301 LAANDLQGCA VATGPYHPIW TGRATDEEPL GLPKCCQPDA ADKASVLEPG
351 RPASAGNALK GRVPPGDSPP GNGSGPRHIN DSPFGTLPGS AEPPLTAVRP
401 EGSEPPGFPT SGPRRRPGCS RKNRTRSHCR LGQAGSGGGG TGDSEGSGAL
451 PSLTCSLTPL GLALVLWTVL GPC
```

Supplementary Fig. 2

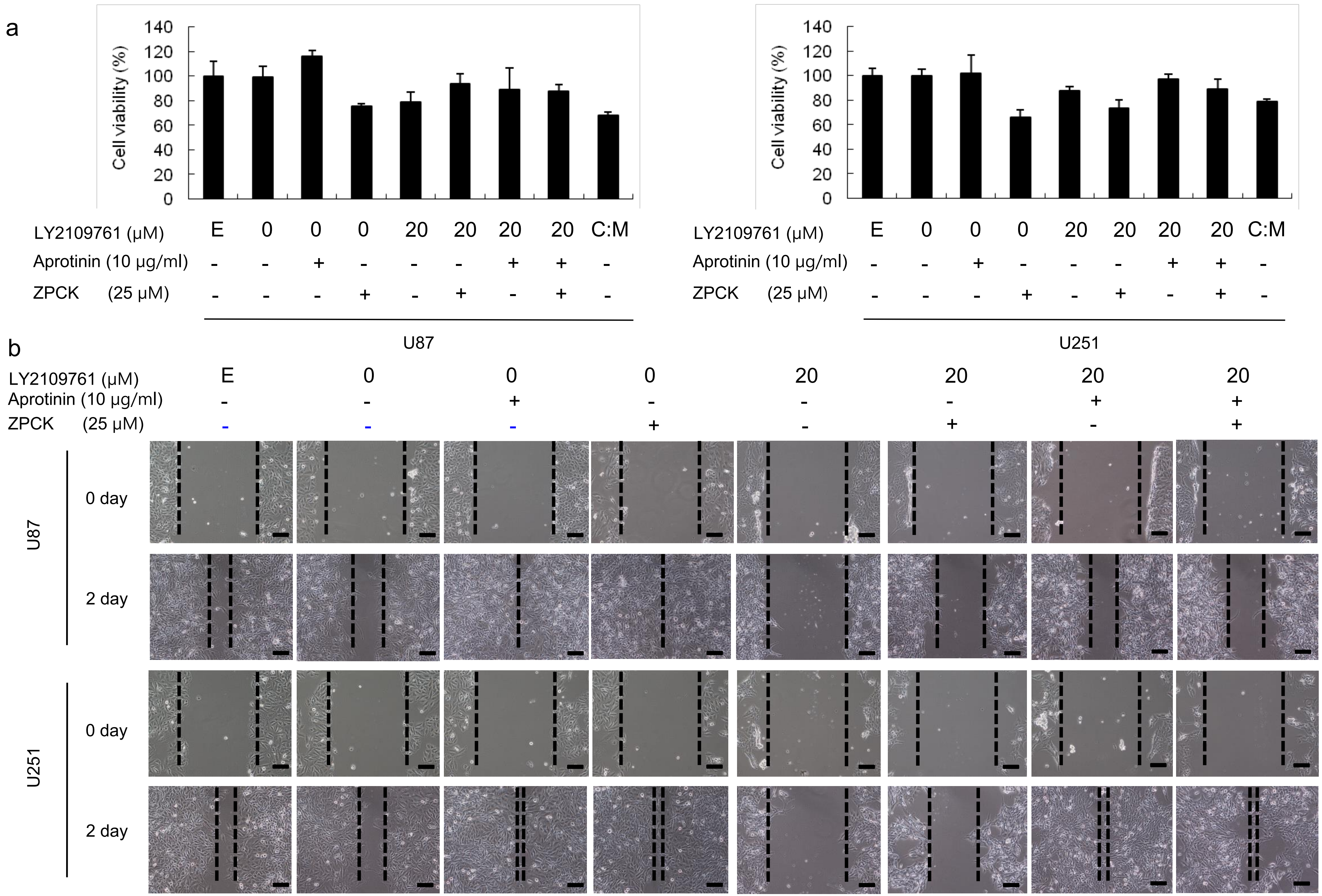

Supplementary Fig. 3

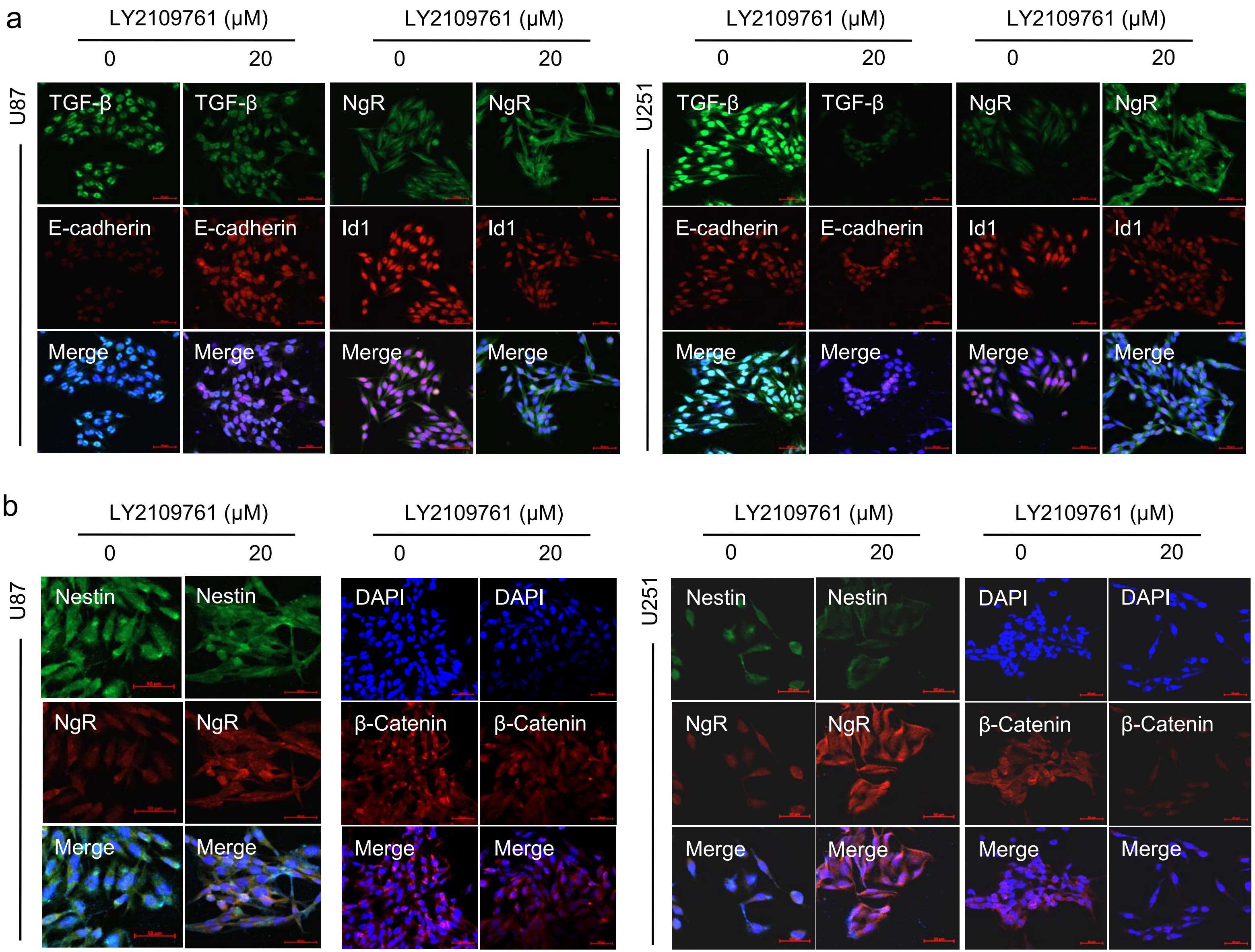

Supplementary Fig. 4

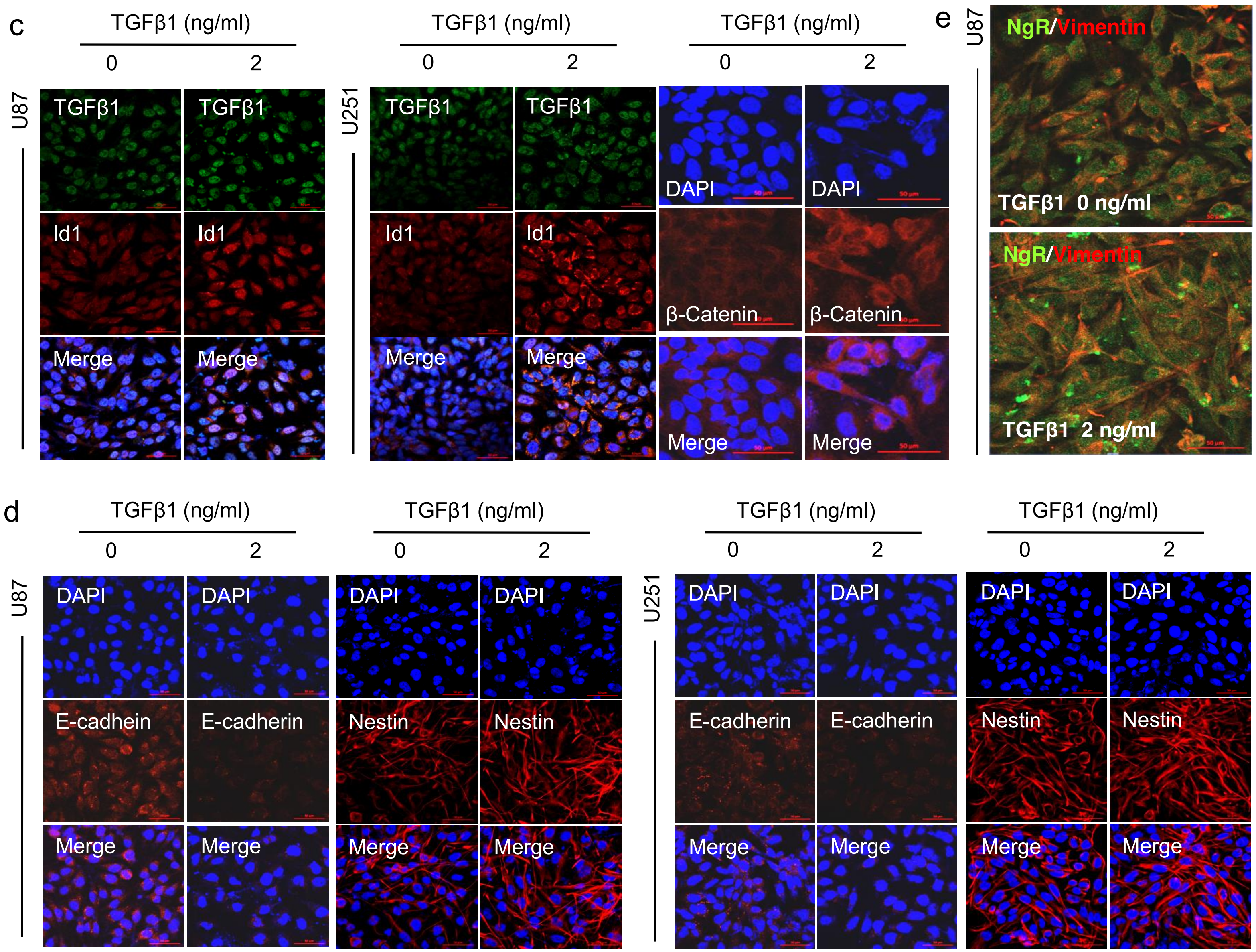

Supplementary Fig. 4 (continued)

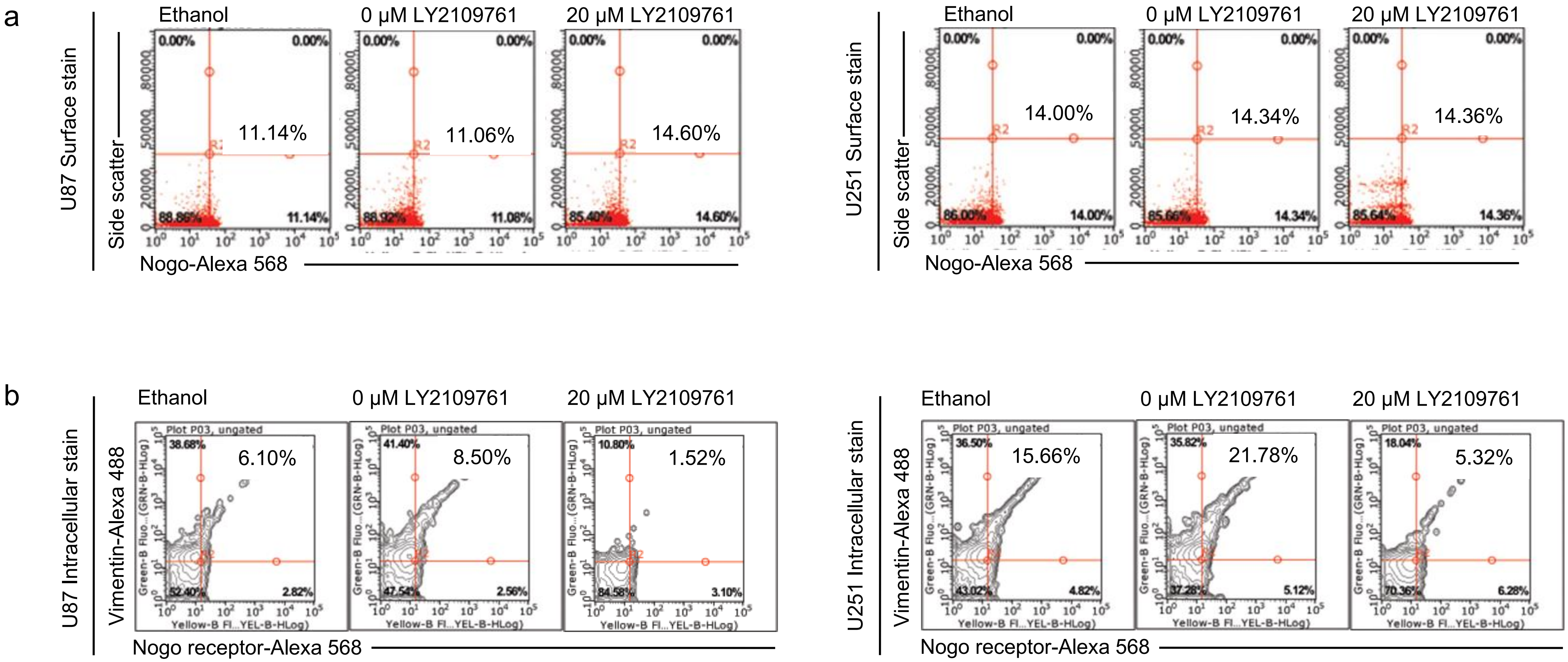

Supplementary Fig. 5

c

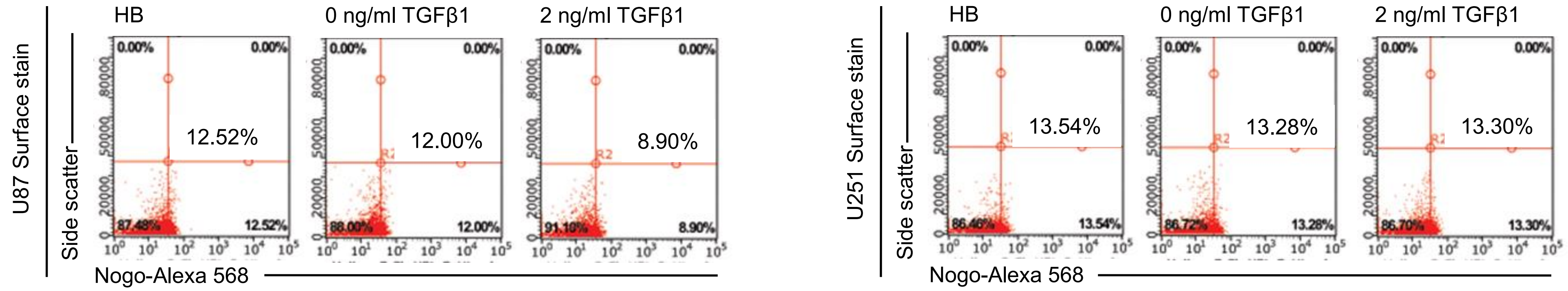

d

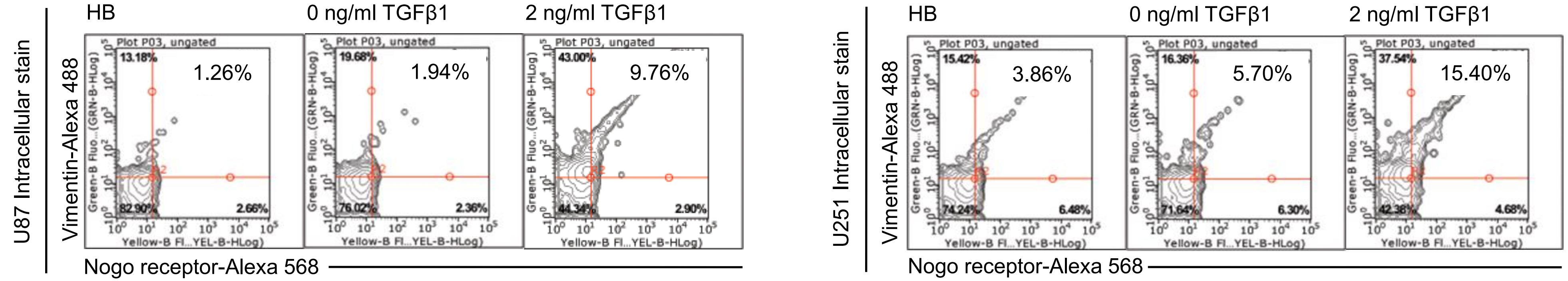

Supplementary Fig. 5 (continued)

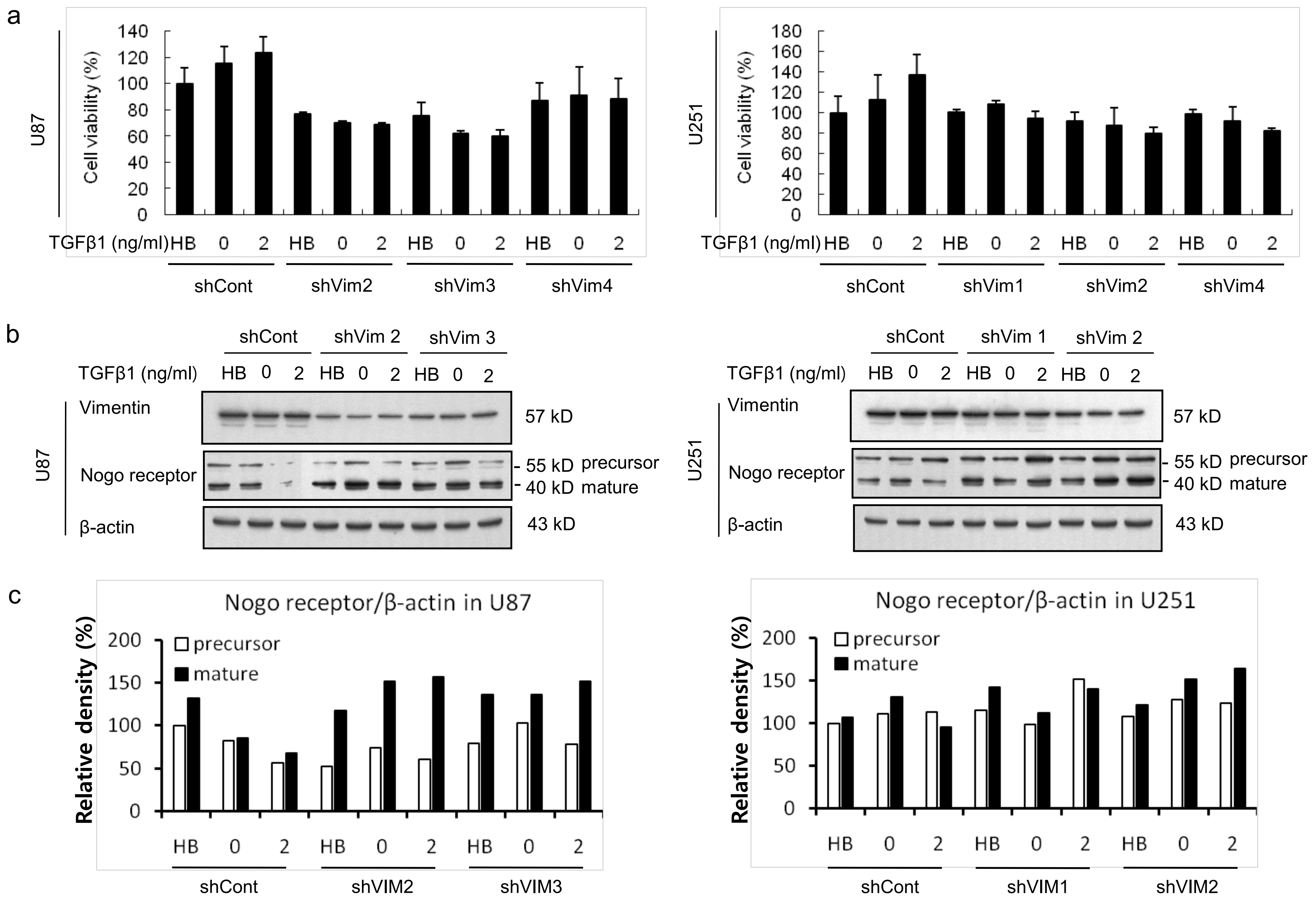

Supplementary Fig. 6

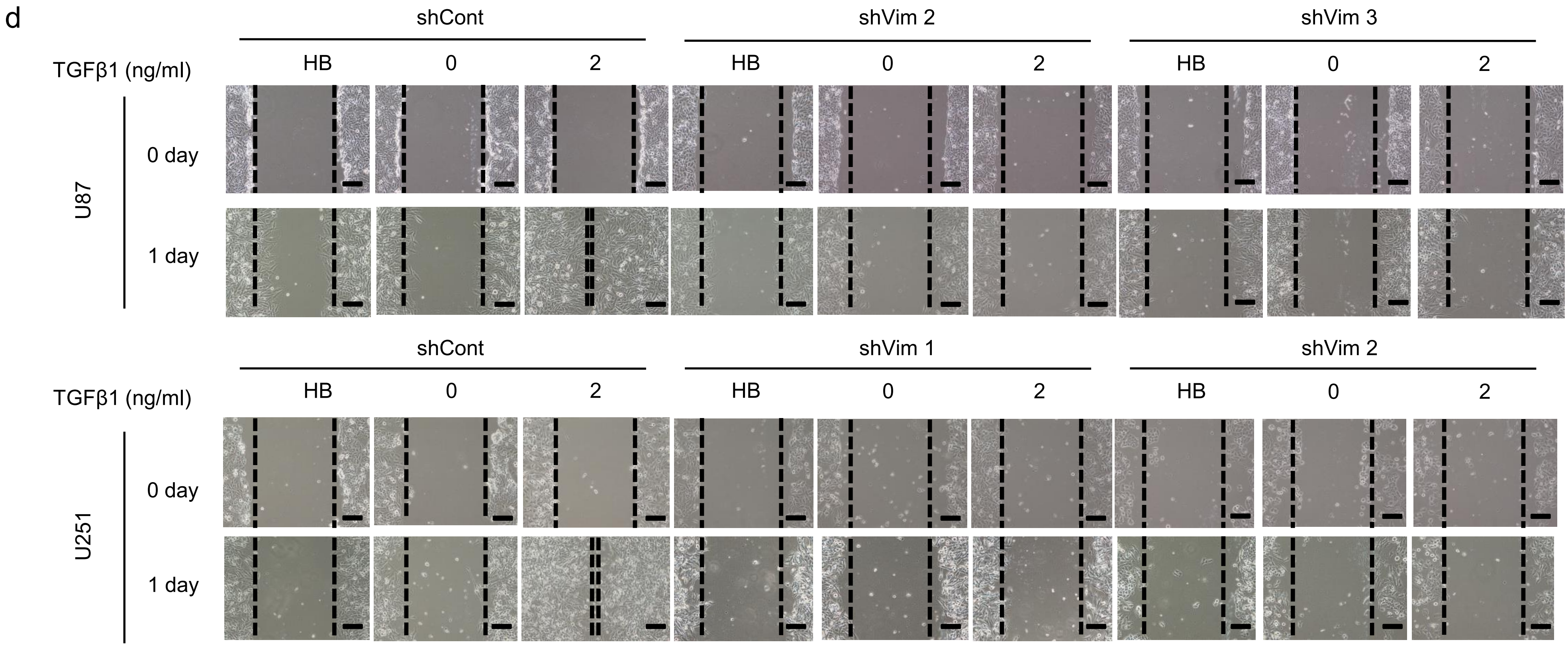

Supplementary Fig. 6 (continued)

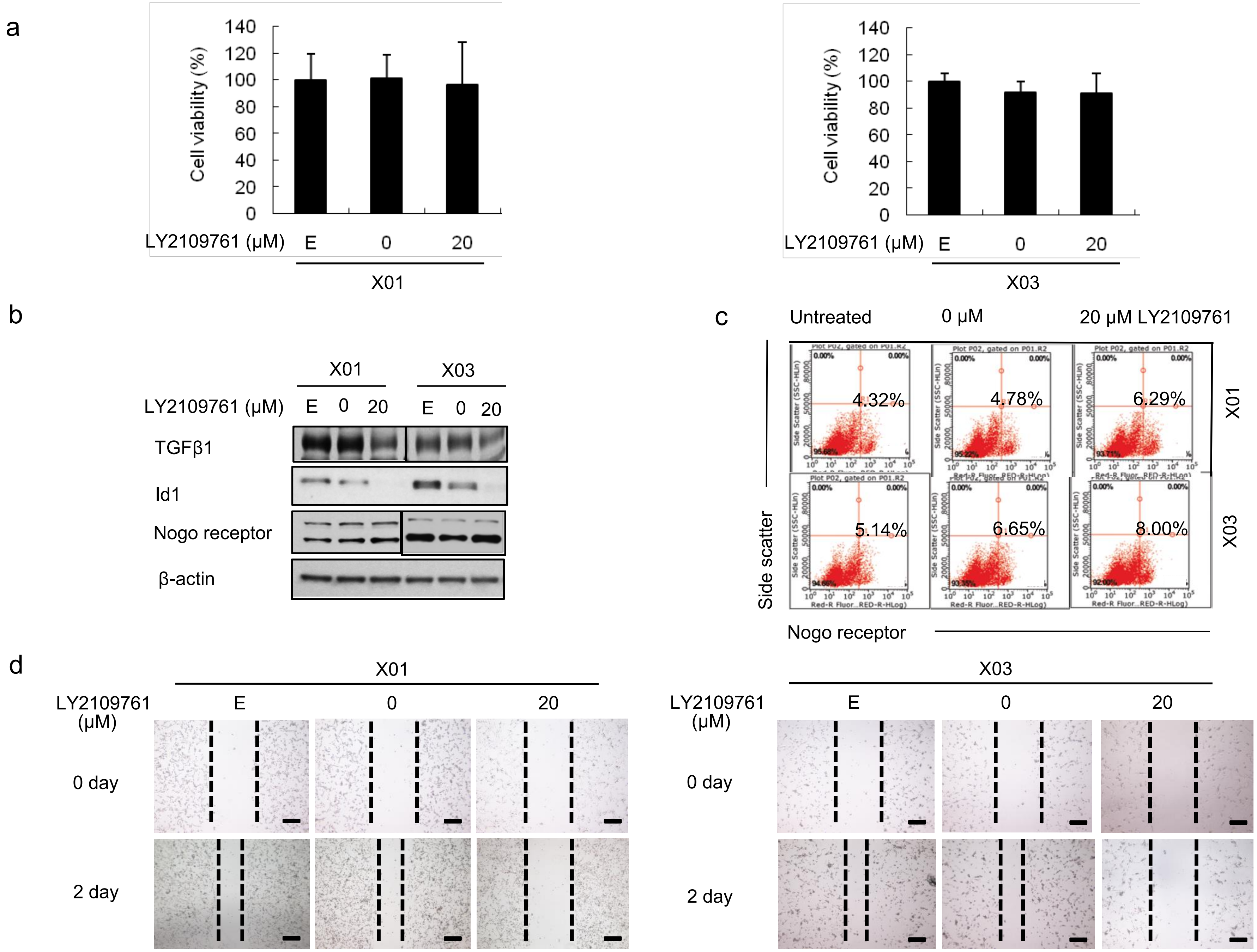

Supplementary Fig. 7

Nogo receptor-vimentin interaction: A novel mechanism for the invasive activity of glioblastoma multiforme

Supplementary Figures legends

Supplementary Fig. 1. Expression of Nogo receptor and Smad family protein in 12 cells including GBM was analyzed by western blot and FACS analysis, respectively. **a, b** Western blotting shows the expression levels of Nogo receptor, phosphorylated-Smad 2, Smad 2, phosphorylated-Smad 3, Smad 3, and Smad 4 in 12 cells. Expression levels of all proteins were normalized to that of β-actin. **c** FACS analysis represents the surface presenting of Nogo receptor. Data are representative of three experiments.

Supplementary Fig. 2. LC-MS/MS analysis of precursor and mature NgR in GBM cells. **a-d** Matched peptide sequences compared with RTN4R database by MASCOT (Matrix Science, http://www.matrix science.com). The LC-MS/MS analysis shows the precursor form in X01 and LY2109761-untreated U251 cells (a and c) and the mature form in C2M and LY2109761-treated U251 cells (b and d).

Supplementary Fig. 3. Effects of carboxypeptidase Y inhibitor (ZPCK and aprotinin) on LY2109761-induced GBM cell migration. **a** In U87 and U251 cells, WST-1 assay reveals that cell viability was not significantly modulated by 20 μM of LY2109761 treatment, 25 μM of ZPCK, 10μg/ml of aprotinin, and in combinations. **b** The scratch wound migration activity of U87 and U251 cells using LY2109761 was decreased by the combinatorial treatment of carboxypeptidase Y inhibitor, ZPCK and aprotinin. U87 and U251 were treated with three drugs as mentioned above for 48 h prior the assay and wound was scratched using 200 μl pipette tip. Duplicate wells were used per condition and three fields per well were captured at each time-point over a period of 48 h. Images of same fields were taken at 0 day and 2 day (x100 magnification). Black scale bar = 100 μm.

Supplementary Fig. 4. The **c**ellular expression and localization of several proteins in GBM cells using LY2109761 or TGF-β1 were determined by laser scanning confocal microscope. Immunofluorescence staining demonstrated that TGFβ inhibition increases Nogo receptor expression in U87 and U251 cells whether activation of TGFβ suppresses the expression of Nogo receptor (a & c). **a** The

treatment of LY2109761 suppressed TGF-β1 and Id1 in both nucleus and cytoplasm, while increasing NgR and E-cadherin in cytoplasm of U87 and U251 cells. **b** Nestin and β-catenin, which was originally localized in cytoplasm, were decreased in LY2109761-treated U87 and U251 cells. **c** The treatment of TGFβ1 slightly increased Id1 and vimentin in cytoplasm of U87 and U251 cells. **d** In the same circumstance, TGFβ1 significantly decreased E-cadherin expression and increased the expression and the translocation of β-catenin in U251 cells. In addition, the expression of Nestin was significantly stronger in TGFβ1- treated U87 and U251 cells than untreated cells. **e** As expected, the colocalization between NgR and vimentin in cytoplasm of U87 cells treated with TGFβ1 was increased. Red scale bar = 50 μm. Data are representative of two experiments.

Supplementary Fig. 5. Interaction of Nogo receptor and vimentin were determined by FACS analysis. **a** Surface staining shows that the inhibition of TGFβ enhances the matured surface Nogo receptor. **b** Intracellular staining reveals that Nogo receptor and vimentin- double positive cells is decreased on LY2109761 treatment. **c** Surface staining shows that the treatment of TGFβ1 decreases the matured surface Nogo receptor. **d** Intracellular staining reveals that Nogo receptor and vimentin-double positive cells is increased in TGFβ1-treated U87 and U251 cells.

Supplementary Fig. 6. Effects of vimentin knockdown on TGFβ1-induced GBM cell migration. GBM cells were transfected with vimentin shRNAs by lentivirus infection. shRNA-transfected U87 and U251 cells were isolated by 3 weeks of puromycin selection, and vimentin expression in each cell line was analyzed by western blotting. β-actin was used as a housekeeping protein control. **a** Vimentin shRNA-transfected GBM cell viability using TGFβ1 was analyzed by WST-1 assay. **b** Western blot analysis shows the expression levels of vimentin and Nogo receptor in vimentin shRNA-transfected U87 and U251 cells using TGFβ1, respectively. **c** Expression levels of precursor and mature Nogo receptor were normalized to that of β-actin. **d** The scratch wound migration activity of the vimentin shRNA-transfected U87 and U251 cells using TGFβ1 was suppressed, while it was enhanced in control shRNA-transfected U87 and U251 cells. Duplicate wells were used per condition and three fields per well were captured at each time-point over a period of 24 h. Images of same fields were taken at 0 day and 1 day (x100 magnification). Black scale bar = 100 μm.

Supplementary Fig. 7. Role of mature NgR on cellular behavior in LY2109761-treated GBMSC. **a** WST-1 assay reveals that the cell viability was not affected by

LY2109761 treatment in X01 and X03 cells. **b** Western blot analysis shows the expression levels of TGFβ1, Id1, and Nogo receptor in LY2109761-treated X01 and X03 cells. Expression levels of all proteins were normalized to that of β-actin. **c** FACS analysis representing the inhibition of TGFβ1 resulting in enhanced matured surface Nogo receptor. **d** The scratch wound migration activity of LY2109761-treated X01 and X03 cells as determined by the scratch wound migration assay. X01 and X03 cells were treated with 20 μM of LY2109761 for 48 h prior to the assay and wound was scratched using a sterile 200 μl pipette tip. Duplicate wells were used for each condition and three fields per well were captured at each time point over a period of 48 h. Images of same fields were taken at day0 and day 2 (100x magnification). Black scale bar = 100 μm. The inhibition of TGFβ1 suppresses the migratory ability of X01 and X03 cells, primary GBMSCs.
